# Worldwide variation in the relative importance of hepatitis B and hepatitis C viruses in hepatocellular carcinoma: a systematic review

**DOI:** 10.1038/sj.bjc.6603649

**Published:** 2007-04-03

**Authors:** S A Raza, G M Clifford, S Franceschi

**Affiliations:** 1 Department of Surgery, The Aga Khan University, Stadium Road, PO Box 3500, Karachi 74800, Pakistan; 2 International Agency for Research on Cancer, 150 cours Albert Thomas, 69372 Lyon cedex 08, France

**Keywords:** hepatitis B virus, hepatitis C virus, hepatocellular carcinoma, meta-analysis

## Abstract

We combined information published worldwide on the seroprevalence of hepatitis B surface antigen (HbsAg) and antibodies against hepatitis C virus (anti-HCV) in 27 881 hepatocellular carcinomas (HCCs) from 90 studies. A predominance of HBsAg was found in HCCs from most Asian, African and Latin American countries, but anti-HCV predominated in Japan, Pakistan, Mongolia and Egypt. Anti-HCV was found more often than HBsAg in Europe and the United States.

Hepatocellular carcinoma (HCC) represents approximately 6% of all new cancer cases diagnosed worldwide, with more than half of these occurring in China alone (Parkin *et al*, 2005). Relatively high incidence rates are also found in South Eastern Asia and in sub-Saharan Africa (Parkin *et al*, 2005). One of the least curable malignancies, HCC is the third most frequent cause of cancer death among men worldwide (Parkin *et al*, 2005).

Chronic infection with hepatitis B virus (HBV) and hepatitis C virus (HCV) are the most important causes of HCC (IARC, 1994). According to the World Health Organisation (WHO), approximately 350 million people are chronically infected with HBV (WHO, 2004) and 170 million with HCV (WHO and the Viral Hepatitis Prevention Board, 1999) worldwide. There are no comparable statistics for the number of individuals coinfected with both HBV and HCV.

The relative importance of HBV and HCV infections in HCC aetiology is known to vary greatly from one part of the world to another (Parkin, 2006), and can change over time (Lu *et al*, 2006). In order to investigate this issue, we collated all published data on the prevalence of chronic HBV and HCV infection among HCC cases.

## MATERIALS AND METHODS

MEDLINE and WHO regional indexed databases were used to search for articles published from 1 January 1989 (after HCV testing became available) to 31 October 2006, by means of the MeSH terms: ‘hepatocellular carcinoma’, ‘hepatitis B virus’ and ‘hepatitis C virus or hepacvirus’. Additional relevant studies were identified in the reference lists of selected articles. No language limitation was imposed. Eligible studies had to report prevalence of both hepatitis B surface antigen (HBsAg) and antibodies against HCV (anti-HCV), alone and in combination, for at least 20 HCC cases. To avoid multiple inclusions of the same HCC cases in more than one article, the time and place of recruitment of cases were cross-checked and the most recent publication was used. In the event that study methods indicated the availability of HBsAg and anti-HCV prevalence data but did not report both of them and the percent of coinfection in the article, authors were contacted for the supplementary information. In the course of contacting authors, additional data became available from one study expanded since the original publication (Appendix A).

The key information extracted from each study were study country, gender distribution, generation of HCV serology tests used, prevalence of HBsAg alone (HBsAg^+^) and anti-HCV alone (anti-HCV^+^) and in combination (HBV/HCV coinfection), and the number of cases that were seronegative for both viral markers.

Key information on 110 selected studies is given in the Appendix A by continent and country. For multicentric studies, HBsAg^+^ and anti-HCV^+^ prevalence data were separated by country (Appendix A). Study size varied substantially and four reports (one each from China, Japan, Taiwan and the United States) included more than 1000 HCC cases. With respect to anti-HCV testing, 17 studies (published from 1989 to 1994) reported the use of first-generation enzyme-linked immunoabsorbant assay (ELISA), 29 studies (published from 1992 to 2003) second-generation ELISA and 42 studies (published from 1997 to 2006) third-generation ELISA. Nineteen studies did not report the generation of HCV testing used; four of these were assumed to have used first-generation ELISA based on date of publication or patient admission. Studies known or likely to have used first-generation ELISA were not included in the computation of HCV prevalence owing to known problems of sensitivity and specificity of those assays (Booth *et al*, 2001). Two studies used HCV RNA instead of anti-HCV, and were included in the analysis (Appendix A).

## RESULTS

**Figure 1:**
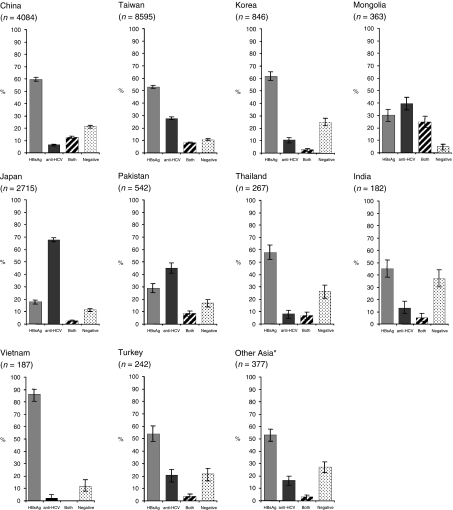
Seroprevalence and corresponding 95% confidence intervals of HBsAg, anti-HCV, both and negative in patients with HCC in Asia. ^*^Indonesia, Myanmar, Iran, Lebanon and Saudi Arabia.

**Figure 2:**
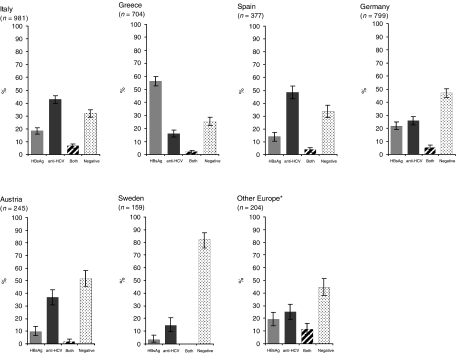
Seroprevalence and corresponding 95% confidence intervals of HBsAg, anti-HCV, both and negative in patients with HCC in Europe. ^*^Belgium and the United Kingdom.

**Figure 3:**
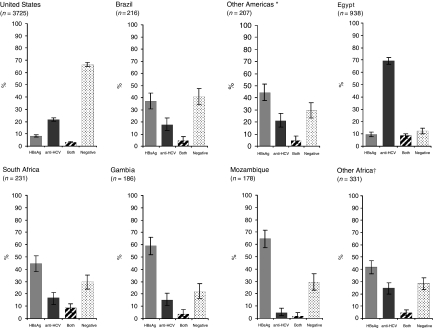
Seroprevalence and corresponding 95% confidence intervals of HBsAg, anti-HCV, both and negative in patients with HCC in the Americas and Africa. ^*^Peru and Mexico; ^†^Sudan, Nigeria, Niger, Senegal and Somalia.

After exclusion of studies using first-generation ELISA for anti-HCV testing, there were 90 studies with relevant data on the prevalence of HBsAg and anti-HCV, covering 27 881 HCC cases from 36 countries (Table 1). The majority of cases were from Asia (66%) followed by the Americas (15%), Europe (12%) and Africa (7%). In Figures 1, 2 and 3, HBsAg^+^ and anti-HCV^+^ prevalence data are shown for countries with information on at least 150 HCC cases. Otherwise countries from the same continent were combined. Substantial variations in HBsAg and anti-HCV prevalence were observed between countries and continents.

### Asia

The largest number of HCC cases from any single country in Asia came from Taiwan, with 8595 HCC cases identified from a single multicentre study (Lu *et al*, 2006), Japan and China (Figure 1). The proportion of HBsAg^+^ HCC cases was greater than 50% in China, Taiwan, Korea, Thailand, Vietnam and Turkey. The lowest proportion of HBsAg^+^ HCC cases was reported in Japan where there was a strong predominance of anti-HCV seropositivity in HCC cases (68%). A higher proportion of anti-HCV^+^ than HBsAg^+^ HCC cases was also found in Pakistan (45%), and in Mongolia (40%), where HBV/HCV coinfection was also very frequent (25%). In China, anti-HCV was found twice as often in combination with HBsAg than alone. The highest proportion of HCC cases seronegative for both hepatitis viruses was found in India (37%).

### Europe

The countries in Europe where the largest numbers of HCC cases were studied were Italy, Greece and Germany (Figure 2). The proportion of HBsAg^+^ HCC cases (56%) was higher than that of anti-HCV^+^ HCC in Greece, whereas the opposite was observed everywhere else in Europe. In Italy and Spain, the proportions of anti-HCV^+^ HCC cases were 43 and 48%, respectively. Seropositivity for anti-HCV was significantly higher than for HBsAg also in Austria and Sweden, whereas in Germany the seroprevalence of the two viruses was similar. Hepatitis B virus/HCV coinfection was rare in most European studies, whereas HCC cases seronegative for both hepatitis viruses were relatively common, measuring over 80% in Sweden.

### The Americas

A majority of American studies on HCC and hepatitis viruses were conducted in the United States (Figure 3), with two-thirds of HCC cases coming from a nation-wide linkage study for the Surveillance Epidemiology and End-Results Program. In the United States, 9% of HCC cases were HBsAg^+^ and 22% were anti-HCV^+^. The prevalence of HBV/HCV coinfection in HCC cases was 3.2% and a high proportion (67%) of HCC cases were seronegative for markers of both hepatitis viruses. In Brazil, 37 and 18% of HCC cases were HBsAg^+^ and anti-HCV^+^, respectively. Only 207 additional HCC cases were available from other American countries (Peru and Mexico), where prevalence of HBsAg exceeded that of anti-HCV.

### Africa

Nearly half of the data on HCC in Africa came from Egypt (Figure 3), where a very high proportion (69%) of HCC cases was anti-HCV^+^. All other African countries showed a preponderance of HBsAg seropositivity. HBV/HCV coinfection did not exceed 10% anywhere in Africa, whereas approximately 30% of HCC cases were seronegative for both hepatitis viruses in South Africa and Mozambique.

## DISCUSSION

This review, based on nearly 30 000 HCC cases, confirms wide international variation in the relative importance of HBV and HCV in this disease. As expected, HBV infection was found substantially more often than HCV infection in HCC cases from the majority of Asian and African countries with the available data. Conversely, more HCC cases were found to be anti-HCV^+^ than HBsAg^+^ in Europe and in the United States, as was also the case in Japan, Pakistan and Mongolia, and in Asia generally. In some countries (i.e., China and Mongolia), more than 10% of HCC cases were coinfected with both hepatitis viruses, thus hampering the attribution of a fraction of HCC cases to HBV or HCV.

More than half of HCC cases were both HBsAg^−^ and anti-HCV^−^ in the United States and some North European countries, thus pointing to the relative importance of heavy alcohol consumption and, possibly, smoking, obesity and diabetes mellitus (Yuan *et al*, 2004) in areas where hepatitis virus prevalence and HCC incidence are low.

Our systematic review failed to identify information on HBV and HCV infection among HCC cases in Eastern Europe, Russia, Central Asia and the majority of African and Latin American countries. None of the studies we found from Oceania using second- or third-generation ELISA met our inclusion criteria. However, a record-linkage study from New South Wales, Australia showed a similar proportion of HBsAg^+^ (45%) and anti-HCV^+^ (53%) HCCs and low frequency of HBV/HCV coinfection (2%) among 281 virus-related HCC cases (Amin *et al*, 2006).

In addition to lack of data from many parts of the world, some weaknesses of our present review should be borne in mind. The extent to which the HCC cases we reported upon are representative, at a national level, is unclear, especially where only small studies were available. Furthermore, important secular trends may be concealed by our analysis, as in the largest study identified (Lu *et al*, 2006), which showed a steady increase in the proportion of HCC cases related to HCV in the last two decades in Taiwan. The vast majority of studies did not provide information on occult HBV infection. Occult HBV infection seems, however, to have little or no clinical significance, at least among immunocompetent individuals (Knoll *et al*, 2006). Most importantly, owing to the long latent period of HCC, seropositivity among HCC cases does not reflect the current importance of the two viruses in the relevant population but rather that two or three decades earlier.

Based upon prevalence of the infections in different populations around the world and a relative risk of 20 for both viruses, Parkin (2006) estimated the fraction of HCC attributable to HBV and HCV in 2002 to be, respectively, 23 and 20% in developed countries and 59 and 33% in developing countries. Our simpler approach, based on HCC cases only, was mainly dictated by the wish to use information from many world populations for whom information on HCC was available but not data on population prevalences of HBV and HCV. It suggests, however, that the relative contribution of HCV to the current HCC burden in middle-aged and old individuals in developed countries and in some developing countries might be higher than in Parkin (2006). In fact, seroprevalence surveys on which attributable risks are based tend to over-sample young individuals at low risk of HCV infection (e.g., blood donors and pregnant women, WHO, 1999; Madhava *et al*, 2002). In conclusion, our findings underline the importance of the prevention of HCV infection that, in the absence of a vaccine, will require an integrated strategy including screening of blood donations, safe injection practices and avoidance of unnecessary injections (Ahmad, 2004).

## Figures and Tables

**Table 1:** Continent-specific distribution of studies with HCC cases^a^

**Continent**	**No. of studies**	**HCC cases**	**Countries represented**
Asia	47	18 400	China, India, Indonesia, Iran, Japan, Korea, Lebanon, Mongolia, Myanmar, Pakistan, Saudi Arabia, Taiwan, Thailand, Turkey and Vietnam
Europe	22	3469	Austria, Belgium, Germany, Greece, Italy, Sweden, Spain and UK
Americas	12	4148	United States, Brazil, Peru and Mexico
Africa	12	1864	Egypt, Gambia, Mozambique, Niger, Nigeria, Senegal, South Africa, Somalia and Sudan
Total	90^b^	27 881	

a Studies that used first-generation ELISA for anti-HCV detection were excluded.

b Total does not add up to 90 owing to three multi-continent studies.

**Table A1:** 

			**Cases**			**Prevalence (%)**			
**First author**	**Reference**	**Country**	**Total**	**Male**	**Female**	**HBsAg^+^**	**Anti-HCV** ^+^	**HBsAg**^+^ **Anti-HCV**^+^	**HBsAg^−^** **anti HCV**^−^
ASIA									
Shi J	*Br J Cancer* 2005; **92**: 607–612	China	3126	—	—	59.6	7.4	14.1	18.9
Ding X^c^	*Jpn J Inf Dis* 2003; **56**: 19–22	China	112	98	14	66.1	2.7	1.8	29.5
Wang BE	*J Med Virol* 2002; **67**: 394–400	China	92	—	—	67.4	4.3	6.5	21.7
Zhang JY	*Int J Epidemiol* 1998; **27**: 574–578	China	152	136	16	55.3	3.3	7.9	33.6
Yu SZ	*Zhonghua Liu Xing Bing Xue Za Zhi* 1997; **18**: 214–216	China	340	—	—	54.1	5.9	12.4	27.6
Yuan JM	*Int J Cancer* 1995; **63**: 491–493	China	76	76	0	64.5	0.0	1.3	34.2
Okuno H	*Cancer* 1994; **73**: 58–62	China	186	168	18	65.6	0.5	4.8	29.0
Tao QM^a^	*Gastroenterol Jpn* 1991; **26**: (Suppl 3) 156–158	China	52	—	—	38.5	9.6	28.8	23.1
Leung NW^a^	*Cancer* 1992; **70**: 40–44	Hong Kong	424	381	43	76.9	3.8	3.5	15.8
Joshi N	*Trop Gastroenterol* 2003; **24**: 73–75	India	40	33	7	47.5	20.0	0.0	32.5
Wang BE	*J Med Virol* 2002; **7**: 394–400	India	15	11	4	26.7	53.3	0.0	20.0
Sarin SK	*J Gastroenterol Hepatol* 2001; **16**: 666–673	India	74	63	11	63.5	4.1	8.1	24.3
Ramesh R	*J Gastroenterol Hepatol* 1992; **7**: 393–395	India	53	45	8	22.6	9.4	5.7	62.3
Wang BE	*J Med Virol* 2002; **67**: 394–400	Indonesia	47	—	—	21.3	40.4	2.1	36.2
Budihusodo U^a^	*Gastroenterol Jpn* 1991; **26** (Suppl 3): 196–201	Indonesia	64	—	—	29.7	50.0	15.6	4.7
Hajiani E	*Saudi Med J* 2005; **26**: 974–977	Iran	71	45	26	52.1	8.5	0	39.4
Ding X^c^	*Jpn J Inf Dis* 2003; **56**: 19–22	Japan	122	88	34	27.9	59.8	9.0	3.3
Sharp GB	*Int J Cancer* 2003; **103**: 531–537	Japan	159	—	—	37.7	24.5	8.8	28.9
Miyazawa K	*Intervirology* 2003; **46**: 150–156	Japan	250	196	54	11.6	80.4	1.2	6.8
Wang BE	*J Med Virol* 2002; **67**: 394–400	Japan	191	140	51	17.8	70.2	1.0	11.0
Tanioka H	*J Infect Chemother* 2002; **8**: 64–69	Japan	1019	709	310	16.4	72.6	0.9	10.1
Fukuhara T	*J Radiat Res* (Tokyo) 2001; **42**: 117–130	Japan	168	—	—	21.4	36.3	11.3	31.0
Koike Y	*Hepatology* 2000; **32**: 1216–1223	Japan	236	164	72	9.7	79.7	0.4	10.2
Abe K	*Hepatology* 1998; **28**: 568–572	Japan	122	89	33	18.0	61.5	4.9	15.6
Tanaka K	*J Natl Cancer Inst* 1996; **88**: 742–746	Japan	91	73	18	18.7	75.8	2.2	3.3
Shiratori Y	*Hepatology* 1995; **22**: 1027–1033	Japan	205	163	42	11.2	83.4	1.0	4.4
Suga M	*Hepatogastroenterology* 1994; **41**: 438–441	Japan	63	51	12	27.0	54.0	9.5	9.5
Eto H	*Southeast Asian J Trop Med Public Health* 1994; **25**: 88–92	Japan	89	69	20	23.6	65.2	3.4	7.9
Kiyosawa K^a^	*Cancer Chemother Pharmacol* 1992; **31**	Japan	162	—	—	13.0	77.8	3.1	6.2
	(Suppl): S150–S156		267	225	42	30.7	59.6	1.5	8.2
			112	94	18	53.6	33.9	4.5	8.0
Yuki N^a^	*Dig Dis Sci* 1992; **37**: 65–72	Japan	148	126	22	17.6	61.5	8.1	12.8
Nishioka K^b^	*Cancer* 1991; **67**: 429–433	Japan	180	—	—	35.6	44.4	6.1	13.9
Saito I^a^	*Proc Natl Acad Sci* 1990; **87**: 6547–6549	Japan	253	207	46	19.4	53.8	0.8	26.1
Ding X^c^	*Jpn J Inf Dis* 2003; **56**: 19–22	Korea	55	42	13	69.1	5.5	3.6	21.8
Kwon SY	*J Gastroenterol Hepatol* 2000; **15**: 1282–1286	Korea	26	—	—	61.5	15.4	0.0	23.1
Abe K	*Hepatology* 1998; **28**: 568–572	Korea	55	42	13	81.8	5.5	3.6	9.1
Shin HR	*Int J Epidemiol* 1996; **25**: 933–940	Korea	170	—	—	65.3	10.0	1.2	23.5
Park BC	*J Viral Hepat* 1995; **2**: 195–202	Korea	540	431	109	58.1	11.3	3.0	27.6
Pyong SJ^a^	*Jpn J Cancer Res* 1994; **85**: 674–679	Korea	90	68	22	15.6	73.3	1.1	10.0
Yaghi C	*World J Gastroenterol* 2006; **2**: 3575–3580	Lebanon	92	78	14	64.1	16.3	3.3	16.3
Tsatsralt-Od B^c^	*J Med Virol* 2005; **77**: 491–499	Mongolia	76	46	30	17.1	14.5	68.4	0
Shizuma T	*Kansenshogaku Zasshi* 2005; **79**: 824–825	Mongolia	90	—	—	34.4	48.9	5.6	11.1
Oyunsuren T	*Asian Pac J Cancer Prev* 2006; **7**: 460–462	Mongolia	197	110	87	30.3	39.7	25.1	5.0
Nakai K	*J Clin Microbiol* 2001; **39**: 1536–1539	Myanmar	25	—	—	56.0	24.0	12.0	8.0
Hamza H	*Proc World Congress of Epidemiology* 2005	Pakistan	57	40	17	21.1	43.9	7.0	28.1
Khokhar N	*J Ayub Med Coll Abbottabad* 2003; **15**: 1–4	Pakistan	57	45	12	15.8	47.4	3.5	33.3
Sharieff S	*Trop Doct* 2001; **31**: 224–225	Pakistan	201	149	52	35.8	41.3	7.0	15.9
Mumtaz MS	*J Rawal Med Coll* 2001; 5: 78–80	Pakistan	44	—	—	25.0	54.5	6.8	13.6
Kausar S	*Pak J Gastroenterol* 1998; **12**: 1–2	Pakistan	30	—	—	16.7	73.3	6.7	3.3
Butt AK	*J Pak Med Assoc* 1998; **48**: 197–201	Pakistan	76	65	11	10.5	75.0	10.5	3.9
Abdul Mujeeb S	*Trop Doct* 1997; **27**: 45–46	Pakistan	54	—	—	42.6	9.3	24.1	24.1
Tong CY	*Epidemiol Infect* 1996; **117**: 327–332	Pakistan	23	22	1	78.3	4.3	4.3	13.0
Ayoola EA	*J Gastroenterol Hepatol* 2004; **19**: 665–669	Saudi Arabia	118	96	22	63.6	8.5	3.4	24.6
Al Karawi MA^a^	*J Gastroenterol Hepatol* 1992; **7**: 237–239	Saudi Arabia	42	38	4	33.3	26.2	4.8	35.7
Khan LA	*Saudi Med J* 2001; **22**: 641–642	Saudi Arabia	24	23	1	20.8	25.0	4.2	50.0
Ozer B	*Turk J Gastroenterol* 2003; **14**: 85–90	Turkey	35	28	7	65.7	28.6	2.9	2.9
Uzunalimoglu O	*Dig Dis Sci* 2001; **46:** 1022–1028	Turkey	207	163	44	52.2	19.3	3.9	24.6
Lu SN	*Int J Cancer* 2006; **119**: 1946–1952	Taiwan	8595	6741	1854	53.2	27.9	8.3	10.7
Tangkijvanich P	*J Gastroenterol* 1999; **34**: 227–233	Thailand	86	69	17	58.1	10.5	8.1	23.3
Tangkijvanich P	*J Gastroenterol* 2003; **38**: 142–148	Thailand	101	86	15	56.4	5.0	8.9	29.7
Songsivilai S	*Trans R Soc Trop Med Hyg* 1996; **90**: 505–507	Thailand	80	—	—	60.0	10.0	3.8	26.2
Ding X^c^	*Jpn J Inf Dis* 2003; **56**: 19–22	Vietnam	38	30	8	60.5	2.6	0	36.8
Cordier S	*Int J Cancer 1993*; **55**: 196–201	Vietnam	149	149	0	92.6	2.0	0	5.4
**Continent subtotal**			**20194**			**48.1**	**29.2**	**7.9**	**14.8**
									
EUROPE									
Schoniger-Hekele M	*Gut* 2001; **48**: 103–109	Austria	245	187	58	9.8	36.7	1.6	51.8
Van Roey G	*Eur J Gastroenterol Hepatol* 2000; **12**: 61–66	Belgium	124	—	—	21.0	23.4	16.9	38.7
Nalpas B^b^	*J Hepatol* 1991; **12**: 70–74	France	47	—	—	6.4	42.6	19.1	31.9
Erhardt A	*Dtsch Med Wochenschr* 2002; **127**: 2665–2668	Germany	192	—	—	21.4	34.9	4.7	39.1
Rabe C	*World J Gastroenterol* 2001; **7**: 208–215	Germany	85	64	21	29.4	24.7	7.1	38.8
Hellerbrand C	*Dig Dis* 2001; **19**: 345–51	Germany	118	94	24	7.6	19.5	0.0	72.9
Kubicka S	*Liver* 2000; **20**: 312–318	Germany	268	214	54	25.0	16.8	10.1	48.1
Petry W	*Z Gastroenterol* 1997; **35**: 1059–1067	Germany	55	—	—	20.0	52.7	0.0	27.3
Goeser T	*Cancer Epidemiol Biomarkers Prev* 1994; **3**: 311–315	Germany	81	66	15	27.2	24.7	1.2	46.9
Raptis I	*J Viral Hepat* 2003; 10: 450–454	Greece	306	265	41	52.3	21.9	0.7	25.2
Kuper HE	*Cancer Causes Control* 2000; **11**: 171–175	Greece	333	283	50	59.5	12.3	3.3	24.9
Hadziyannis S	*Int J Cancer* 1995; **60**: 627–631	Greece	65	49	16	56.9	7.7	4.6	30.8
Kaklamani E^a^	*JAMA* 1991; **265**:1974–1976	Greece	185	166	19	22.7	15.7	23.2	38.4
Franceschi S	*Cancer Epidemiol Biomarkers Prev* 2006; **15**: 683–689	Italy	229	183	46	10.0	61.1	3.9	24.9
Donato F	*Oncogene* 2006; **25**: 3756–3770	Italy	583	—	—	19.7	37.9	2.7	39.6
Ricci G	*Cancer Lett* 1995; **98**: 121–125	Italy	104	—	—	31.7	20.2	34.6	13.5
Stroffolini T	*J Hepatol* 1992; **16**: 360–363	Italy	65	47	18	16.9	58.5	7.7	16.9
Simonetti RG^a^	*Ann Intern Med* 1992; **116**: 97–102	Italy	212	161	51	7.1	62.7	8.5	21.7
Levrero M^b^	*J Hepatol* 1991; **12**: 60–63	Italy	167	135	32	22.8	49.1	9.0	19.2
Colombo M^a^	*Lancet* 1989; **2**: 1006–1008	Italy	132	115	17	14.4	48.5	16.7	20.5
Ladero JM	*Eur J Cancer* 2006; **42**: 73–77	Spain	184	150	34	4.9	63.0	1.6	30.4
Rodriguez Vidigal FF	*An Med Interna* 2005; **22**: 162–166	Spain	42	37	5	11.9	42.9	0.0	45.2
Ding X^c^	*Jpn J Inf Dis* 2003; **56**: 19–22	Spain	57	45	12	38.6	12.3	3.5	45.6
Crespo J	*Med Clin (Barc)* 1996; **106**: 241–245	Spain	94	82	12	18.1	43.6	10.6	27.7
Bruix J^a^	*Lancet* 1989; **2**: 1004–1006	Spain	96	67	29	4.2	69.8	5.2	20.8
Widell A	*Scand J Infect Dis* 2000; **32**: 147–152	Sweden	95	—	—	5.3	16.8	0	77.9
Kaczynski J	*Scand J Gastroenterol* 1996; **31**: 809–813	Sweden	64	48	16	0	10.9	0	89.1
Haydon GH	*Gut* 1997; **40**: 128–132	United Kingdom	80	—	—	16.3	27.5	2.5	53.8
**Continent subtotal**			**4308**			**23.1**	**34.3**	**6.5**	**36.1**
									
AFRICA									
Ezzat S^d^	*Int J Hyg Environ* Health 2005; **208**: 329–339	Egypt	450	—	—	3.8	82.0	5.3	8.9
Abdel–Wahab M	*Hepatogastroenterology* 2000; **47**: 663–668	Egypt	385	—	—	14.5	61.0	7.0	17.4
Hassan MM	*J Clin Gastroenterol* 2001; **33**: 123–126	Egypt	33	23	10	12.1	72.7	3.0	12.1
Darwish MA	*J Egypt Public Health Assoc* 1993; **68**: 1–9	Egypt	70	57	13	21.4	30.0	40.0	8.6
Kirk GD	*Hepatology* 2004; **39**: 211–219	Gambia	186	—	—	59.1	15.1	3.8	22.0
Dazza MC	*Am J Trop Med Hyg* 1993; **48**: 237–242	Mozambique	178	141	37	64.6	4.5	1.7	29.2
Cenac A	*Am J Trop Med Hyg* 1995; **52**: 293–296	Niger	26	19	7	57.7	7.7	15.4	19.2
Olbuyide IO	*Trans R Soc Trop Med* Hyg 1997; **91**: 38–41	Nigeria	64	42	22	48.4	7.8	10.9	32.8
Kew MC	*Gastroenterology* 1997; **112**: 184–187	South Africa	231	201	30	44.6	16.9	8.7	29.9
Ka MM	*Dakar Med* 1996; Spec No: 59–62	Senegal	64	56	8	34.4	64.1	1.6	0
Bile K	*Scand J Infect Dis* 1993; **25**: 559–564	Somalia	62	53	9	37.1	35.5	4.8	22.6
Omer RE	*Trans R Soc Trop Med Hyg* 2001; **95**: 487–491	Sudan	115	88	27	41.7	10.4	0.9	47.0
**Continent subtotal**			**1864**			**30.0**	**43.2**	**6.8**	**20.0**
									
NORTH AMERICA									
Marrero JA	*J Hepatol* 2005; **42**: 218–224	United States	70	44	26	7.1	51.4	0.0	41.4
Davila JA	*Gastroenterology* 2004; **127**: 1372–1380	United States	2584	1721	863	5.8	13.3	2.9	77.9
Ding X^c^	*Jpn J Inf Dis* 2003; **56**: 19–22	United States	65	41	24	15.4	41.5	3.1	40.0
Hassan MM	*Hepatology* 2002; **36**: 1206–1213	United States	115	87	28	11.3	19.1	3.5	66.1
Abe K	*Hepatology* 1998; **28**: 568–572	United States	65	40	25	10.8	41.5	1.5	46.2
Yu MC	*Hepatology* 1997; **25**: 226–228	United States	111	67	44	7.2	31.5	1.8	59.5
Nomura A	*J Infect Dis* 1996; **173**: 1474–1476	United States	24	24	0	62.5	0.0	0.0	37.5
Di Bisceglie	*Am J Gastroenterol* 2003; **98**: 2060–2063	United States	691	—	—	15.5	46.6	4.8	33.1
Di Bisceglie^a^	*Am J Gastroenterol* 1991; **86**: 335–338	United States	99	67	32	6.1	12.1	1.0	80.8
Hasan F^a^	*Hepatology* 1990, **12**: 589–591	United States	87	—	—	27.6	35.6	4.6	32.2
**Continent subtotal**			**3911**			**8.8**	**21.9**	**3.1**	**66.1**
									
LATIN AMERICA									
Miranda EC	*Rev Soc Bras Med Trop* 2004; **37** (Suppl 2): 47–51	Brazil	36	31	5	58.3	0.0	8.3	33.3
Goncalves CS	*Rev Inst Med Trop Sao Paulo* 1997; **39**: 165–170	Brazil	180	139	41	32.8	21.1	3.9	42.2
Mondragon Sanchez R	*Hepatogastroenterology* 2005; **52**: 1159–1162	Mexico	71	—	—	8.5	60.6	14.1	16.9
Ruiz E	*Rev Gastroenterol Peru* 1998; **18**: 199–212	Peru	136	116	20	63.2	0.7	0.0	36.0
**Continent subtotal**			**423**			**40.7**	**19.4**	**4.7**	**35.2**
									
OCEANA									
Yip D^b^	*World J Gastroenterol* 1999; **5**: 483–487	Australia	63	43	20	28.6	3.2	4.8	63.5
**Total**			**30763**			**38.3**	**29.7**	**7.0**	**25.0**

a Studies reporting first generation ELISA.

b Studies presumed to have used first-generation ELISA.

c Studies reporting only HCV RNA testing.

d Data has been expanded since original publication.

## Appendix A - See over

Table A1
